# Crystal Structure of the *Salmonella* Typhimurium Effector GtgE

**DOI:** 10.1371/journal.pone.0166643

**Published:** 2016-12-06

**Authors:** Caishuang Xu, Guennadi Kozlov, Kathy Wong, Kalle Gehring, Miroslaw Cygler

**Affiliations:** 1 Department of Biochemistry, University of Saskatchewan, Saskatoon, Saskatchewan, Canada; 2 Department of Biochemistry, Groupe de recherche axé sur la structure des protéines, McGill University, Montreal, Quebec, Canada; George Washington University, UNITED STATES

## Abstract

*Salmonella* Typhimurium GtgE is an effector protein contributing to the virulence of this pathogen. It was shown to possess highly selective proteolytic activity against a subset of Rab proteins that helps in evasion of *Salmonella*-containing vacuole (SCV) fusion with lysosomes. Cys45, His151 and Asp169 are essential for proteolytic activity. The structure of a C-terminal fragment GtgE(79–214) indicated the presence of a papain-like fold. Here, we present the structure of GtgE(17–214) containing the fully assembled active site. The design of a proteolytically active and crystallizable GtgE construct was aided by NMR spectroscopy. The protein indeed displays papain-like fold with an assembled Cys-His-Asp catalytic triad. Like the full-length GtgE, the crystallizable construct showed low activity *in vitro* for its known substrates, Rab32 and Rab29. NMR titration experiments showed at most very weak binding of GtgE to the peptide encompassing the Rab29 cleavage site. In view of the low *in vitro* activity and poor substrate binding, we postulate that the function of GtgE *in vivo* as a proteolytic enzyme is dependent on other factor(s), such as a protein partner or interactions with the SCV membrane, which stimulate(s) GtgE activity *in vivo*.

## Introduction

Phagocytosis is an important defense mechanism for host cells in fighting against bacterial infection [1]. The phagosome, acidified through the action of vacuolar ATPase in the phagosome membrane, is directed to fuse with the lysosome, which releases anti-microbial proteins to destroy the invader [1, 2]. The protein markers Rabs (Ras-related in brain) GTPases, Rab8B, Rab13 and Rab35, are classical markers of the phagosome membrane and promote the fusion of the phagosome to the lysosome [2]. These Rab GTPases belong to the Ras superfamily of small GTPases [3] and play essential roles in regulation of membrane identity, vesicle formation, vesicle and organelle motility and vesicular trafficking [4–6]. Since Rabs regulate phagosome dynamics [7], it is not surprising that pathogens target them with effector proteins to perturb their function.

Unlike the classic phagosome, the *Salmonella*-containing vacuole (SCV) avoids fusion with lysosome through a distinct pathway [8]. The maturation of SCV involves the recruitment of a different set of small GTPases, Rab5, Rab7 and Rab11 [9]. Upon formation of SCV, *Salmonella* secrets a number of effector proteins through the type 3 secretion system (T3SS). The effector SopB engages Rab5 at the SCV membrane [10]. Subsequently, Rab5 attracts a phosphatidylinositol 3-kinase Vps40, which generates phosphatidylinositol 3-phosphate (PI3P) and blocks phago-lysosomal fusion [10]. PI3P in turn attracts the early endosome-associated protein EEA1, which also binds Rab5 [1, 10]. SopB recruits the sorting nexin1 protein, which removes the late-endosomal marker mannose 6-phosphate receptor from the membrane [11]. SopB also promotes activation of Rab14, which delays the SCV-lysosome fusion and facilitates bacterial replication inside the SCV [12].

Maturation of the SCV also requires SopD2, which interacts with a late endosome protein marker Rab7, although bacteria try to limit the interaction between SCV and the late endosome [13]. Rab7 attracts Rab-interacting lysosomal protein (RILP), which bridges the Rab7-containing membrane with the microtubule dynein motor complex [14, 15]. With the help of Rab7 and RILP, the SCV traffics along the microtubules. Several effectors, such as SifA, SopA, SopD2, SspH2, and SsaB, are involved in mediating the SCV-associated actin dynamics and the formation of *Salmonella* induced filaments, which are essential for SCV trafficking [2, 8, 14, 16].

In *S*. Typhimurium, which infects a broad range of hosts, another effector, GtgE, plays an essential role in virulence. Strains lacking this prophage-borne effector show attenuated virulence [17]. GtgE is encoded within the Gifsy-2 lambdoid prophage. Of the ten putative virulence genes encoded in the Gifsy-2 prophage, the major virulence determinants are the g*tgE* and *sodCI* genes [18].

Interestingly, *gtgE* is absent in *S*. Typhi [19], which only infects humans. *S*. Typhi produces a unique virulence protein, typhoid toxin [20]. Typhoid toxin is expressed exclusively during intracellular growth and is transported to the extracellular environment by vesicular transport [21]. Rab GTPases are usually involved in vesicular trafficking and it was found that deletion of Rab29 decreased the amount of typhoid toxin transport intermediates [19]. Rab29 was further characterized to be present on the SCV membrane of *S*. Typhi, but not on the SCV membrane of *S*. Typhimurium [19]. The differential recruitment of Rab29 on SCVs was traced to the presence of GtgE [19]. *S*. Typhi complemented with *gtgE* propagated more efficiently within the SCV than the wild-type strain in human macrophages and showed significantly increased survival ability in primary bone-marrow-derived macrophages from mice, a non-permissive species [19]. This indicates that the expression of the *S*. Typhimurium effector GtgE in *S*. Typhi permitted *S*. Typhi to overcome the host restriction barrier.

Recently GtgE was shown to be a cysteine protease with active site consisting of Cys45, His151 and Asp169 [22]. It was previously shown to cleave Rab29 in *in vitro* experiments [19]. The crystal structure of a fragment of GtgE containing residues 80–213 was determined and its fold confirmed to be typical of papain-like cysteine protease clan CA [22]. Although the structure of GtgE^(80–213)^ aligns well with the other peptidases from clan CA, the orientations of His151 and Asp169 are different with respect to the catalytic residues histidine and aspartate in proteins from this clan. This difference in orientation may arise from the absence of the N-terminal region of GtgE, which includes Cys45. As a result, the crystallized GtgE fragment represents an inactive conformation.

Here, we have delineated the minimal fragment necessary for enzymatic activity and determined the structure of the active form of GtgE. NMR spectroscopy was used to identify mobile regions of this protein. Deletion of one of these regions led to the successful crystallization of active GtgE. The structure shows that Cys45 indeed interacts with His151 and Asp169 to form an active site. This site is not fully assembled in the absence of substrate and rationalizes the low activity observed in *in vitro* experiments.

## Experimental Methods

### Constructions of GtgE and Rab32 expression plasmids

Target gene GtgE (*S*. Typhimurium GtgE, NCBI reference NP_460029.1) fragment 2–228 was inserted into pMCSG7 vector as an N-terminal His-tagged fusion. The GtgE fragments 2–228, 14–214, 31–214 and Rab32 (human, NCBI reference NP_006825.1) 2–225 fragment were cloned into pRL652 vector as GST-tagged fusions. These plasmids were constructed by ligase-independent method [23]. The GtgE fragments 17–214, 79–214 and 14–79 containing the C45S mutation were cloned into pET29a vector as C-terminal His-tagged fusions. The MBP-Rab29 plasmid was provided by Dr. Stefania Spanò, University of Aberdeen, UK.

### Mutagenesis of GtgE

His-GtgE(2–228) plasmid was amplified with primers carrying His151Ser mutation and GST-GtgE(14–214) plasmid was amplified with primers carrying Cys45Ser mutation. After amplification, the DNA mixture was treated with DpnI at 37°C for 2 h and then DpnI was inactivated at 80°C for 20 min. The DNA mixture was transformed into DH5α component cell, then plated on LB-agar supplemented with 100 mg/mL ampicillin. Mutagenesis to obtain GtgE(17–214, Δ33–40) and GtgE(17–214, Δ59–68) was then performed using QuickChange Lightning Site-Directed Mutagenesis Kit (Agilent Technologies). Extracted plasmids were sequenced by Eurofins MWG Operon LLC.

### Expression and purification of the His-tagged GtgE constructs

Plasmids carrying His-tagged GtgE were transformed into BL21(DE3) cell line, plated on LB-agar. Ampicillin at the concentration of 100 mg/mL (MCSG7) or kanamycin at the concentration of 30 mg/mL (pET29a) were used for selection. Single colony was inoculated in 20 mL LB medium, which was incubated in a 37°C shaker for overnight. The overnight culture was inoculated into 1 L TB medium, growing at 37°C. When OD_600_ reached 1.2, cell culture was induced with 0.5 mM ITPG and transferred into a 20°C shaker for 16 h. After expression, cell culture was spun down at 7000×g for 20 min and re-suspended in 50 mL lysis buffer (50 mM HEPES pH 7.5, 500 mM NaCl, 5% glycerol and 5 mM imidazole), supplemented with 1 mM benzamidine. The cells were lysed in a cell disrupter (Constant Systems Ltd, TS Series Benchtop). The cell lysate was centrifuged at 30,000×g for 30 min. The supernatant was loaded to nickel-nitrilotriacetic acid (Ni-NTA) agarose resin (Qiagen, Valencia, CA, USA), which was pre-equilibrated with lysis buffer. The protein-resin mixture was incubated at 4°C for 30 min. After binding, resin was washed with 5 column volumes of lysis buffer and then with 2 column volumes of 30 mM imidazole solution. Subsequently, the His-tagged protein was eluted with 500 mM imidazole. Eluted protein was concentrated and loaded onto size exclusion column (Superdex 75 10/300 GL column, GE or ENrich SEC70 10×300 column, Bio-Rad), which was pre-equilibrated with buffer containing 20 mM HEPES pH 7.5, 100 mM NaCl. Purified protein was also analyzed by size-exclusion chromatography-multi-angle light scattering (SEC-MALS) (WTX030S5-miniDAWN TREOS-Optilab, Wyatt Technologies Corporation, Santa Barbara, CA) to determine the molecular weight distribution and homogeneity in solution.

### Expression and purification of the GST-tagged GtgE constructs

The expression protocol was the same as described for expression His-tagged GtgE except using BL21 strain. The cell culture was spun down and the cells were re-suspended in 50 mL lysis buffer (15 mM HEPES pH 7.5, 150 mM NaCl, 1 mM DTT), supplemented with 1 mM benzamidine. After lysis of cell, the cell lysate supernatant was transferred to incubate with glutathione (GSH) agarose resin, pre-equilibrated with lysis buffer. The protein solution and GSH resin was incubated at 4°C for 1 h for binding. The resin was washed with 3 column volumes of lysis buffer. The GST-tagged protein bound to the GSH resin was treated with TEV protease in a ~1:50 (w/w) ratio in 10 ml of lysis buffer at room temperature for 12 h. After the cleavage reaction, the protein solution was loaded onto a Ni-NTA column to remove TEV protease. The cleaved protein was concentrated and loaded to size exclusion column (Superdex 75 10/300 GL column, GE or ENrich SEC70 10×300 column, Bio-Rad). The HPLC buffer used was 20 mM HEPES pH 7.5, 100 mM NaCl.

### Crystallization trials

GtgE constructs were purified and concentrated to approximately 20 mg/mL. Crystallization trials were carried out on GtgE mutants in the absence and presence of peptides corresponding to the known Rab32 cleavage site. The screens were performed in 96-well plates in a sitting drop format using the Gryphon crystallization robot (Art Robbins Instruments, Sunnyvale, CA, USA) and 24-well plates in a hanging drop format. Micro-batch crystallization approach was also used to set up screens. Crystallization screening was performed using both commercial (Hampton Research, Aliso Viejo, CA, USA and Qiagen, Valencia, CA, USA) and home-made incomplete factorial screens. Promising conditions were found only for the GtgE(17–214, Δ33–40, C45S) fragment and they were explored further by systematic modifications of the initial conditions within a narrow range. The best crystals for GtgE(17–214, Δ33–40, C45S) were obtained at 20°C by equilibrating a 0.8 μL drop of a protein concentrated to 49 mg/mL in 10 mM HEPES pH 7.0, 0.1 M NaCl, 2 mM DTT, mixed with 0.8 μL of reservoir solution containing 1.7–1.85 M ammonium sulfate, 0.1 M Bis-Tris (pH 5.5) and 10–25% (w/v) glycerol and suspended over 1 mL of reservoir solution. For data collection, crystals were picked up in a nylon loop and flash cooled in a N_2_ cold stream (Oxford Cryosystem).

### Structure determination and refinement

The native dataset from a GtgE crystal was collected using a single-wavelength (0.63 Å) regime on an ADSC Quantum-210 CCD detector (Area Detector Systems Corp.) at beamline A1 at the Cornell High-Energy Synchrotron Source (CHESS) (Table 1). Data processing and scaling were performed with HKL2000 [24]. The starting phases were obtained using molecular replacement with the GtgE(79–214) structure (PDB code 4MI7) using PHASER [25]. The starting model was extended by program ARP/wARP [26], which automatically built part of the N-terminal region of GtgE. This model was further extended manually using program Coot [27] and was improved by several cycles of refinement using the program REFMAC [28]. The final model has good stereochemistry with no outliers in the Ramachandran plot computed using PROCHECK [29]. Coordinates have been deposited in the RCSB Protein Data Bank (http://www.rcsb.org) with accession code 5KDG (http://www.rcsb.org/pdb/explore/explore.do?structureId=5KDG).

**Table 1:** Statistics of data collection and refinement.

	GtgE
**Data collection**	
Space group	P4_3_2_1_2
*a*, *b*, *c* (Å)	64.30, 64.30, 124.21
Resolution (Å)^1^	50–1.73 (1.76–1.73)
*R*_sym_^1^	0.042 (0.684)
*I* / ơ *I*^1^	37.8 (2.7)
Completeness (%)^1^	99.6 (100.0)
Redundancy^1^	6.7 (6.9)
CC1/2^1^	0.99 (0.81)
**Refinement**	
Resolution (Å)	57.1–1.73
No. reflections	26504
*R*_work_ / *R*_free_	0.209/0.238
No. atoms	1571
Protein	1507
Water	64
*B*-factors	
Protein	21.4
Water	41.8
R.m.s deviations	
Bond lengths (Å)	0.007
Bond angles (°)	1.01
Ramachandran statistics (%)	
Most favored regions	98.9
Additional allowed regions	1.1
PDB code	5KDG

^1^ Highest resolution shell is shown in parentheses.

### Protease activity of GtgE constructs

Activity of the recombinant GtgE and mutant proteins was monitored by following the cleavage of GST-Rab32 following described protocol [19]. GtgE cleaves Rab32 after Gly59 [30] resulting in a ~35 kDa GST-Rab32(1–59) fragment. The appearance of this fragment was followed by SDS-PAGE

GST-Rab32 was purified in a similar fashion to GST-tagged GtgE, except for omitting the GST tag cleavage step. GST-Rab32 was eluted with 20 mM GSH in buffer containing 15 mM HEPES pH 7.5, 150 mM NaCl and 1 mM DTT. Eluted GST-Rab32 was dialysis against buffer containing 15 mM HEPES pH 7.5, 30 mM NaCl and 1 mM DTT at 4°C for 16–20 h. After dialysis, the protein was loaded onto an anion exchange HitrapQ column, which was pre-equilibrated with buffer A, composed of 15 mM HEPES pH 7.5 and 1 mM DTT. After loading the protein, the column was washed with 2 column volumes of buffer A. Protein was eluted with a salt step gradient in buffer A, which salt steps of 50 mM, 100 mM, 200 mM, 300 mM, 500 mM and 1M NaCl. Fractions were collected and analyzed by SDS-PAGE.

Each GtgE construct was added to solution containing GST-Rab32 at 40 μM concentration. The reaction was carried at room temperature with GtgE:GST-Rab32 molar ratio 1:2 or 1:20, in the presence of 10 mM Mg^2+^ and 10 mM Ca^2+^. GST-Rab32 alone under the same conditions served as a negative control. An aliquot of the reaction mixture was removed after 1 h and 2 h incubation. The reaction was terminated by adding the SDS-PAGE loading buffer and by denaturing the proteins at 95°C for 10 min. The samples were analyzed by SDS-PAGE.

### NMR spectroscopy

Partial NMR resonance assignments of GtgE(17–214) were carried out using 0.5 mM ^13^C,^15^N-labeled protein samples in 20 mM MES (pH 6.5), 50 mM NaCl at 308 K using Varian 500 MHz and 800 MHz spectrometers. Protein signal assignments were performed using standard techniques, including three-dimensional experiments HNCA, HN(CO)CA, HNCO, HN(CA)CO, HNCB, NH(COCA)CB. For NMR titrations, unlabeled RATIGVDFALK peptide or MBP-fused Rab29 were added to the ^15^N-labeled 0.2 mM GtgE(17–214) to the final molar ratio of 5:1 or 1:1, respectively. All NMR titrations were performed at 303 K using Bruker 600 MHz spectrometer. NMR spectra were processed using NMRPipe [31] and analyzed with NMRView [32].

## Results and Discussion

### Crystallization of GtgE

GtgE is a cysteine protease and contains a Cys-His-Asp catalytic triad [19, 22, 30]. The critical base was identified as His151 [19], the nucleophile as Cys45 and the third residue as Asp169 [22]. Previous attempts to crystallize GtgE met with partial success and led to the crystal structure of the 79–214 fragment [22]. Initially, we have made several truncated constructs, all of which included the catalytic triad residues, namely 2–228, 14–214, 17–214 and 31–214. Enzymatic assays showed that truncation of up to 17 N-terminal residues does not affect activity, the removal of 31 residues from the N-terminus renders the protein inactive despite containing the intact triad. Extensive crystallization screening of the first three constructs did not lead to crystals.

**Fig 1:**
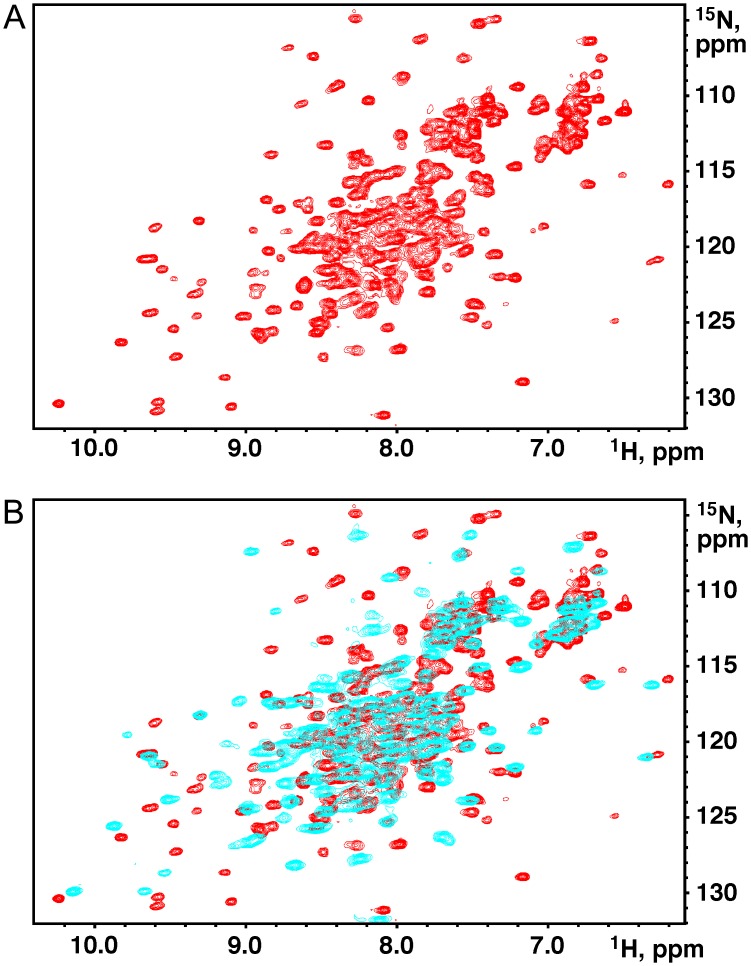
^1^H,^15^N-correlation HSQC spectra of GtgE. (A) GtgE(17–214), dispersion of peaks is characteristic of a well-folded protein. (B) Superposition of HSQC spectra of GtgE(17–214) (red) and GtgE(79–214) (cyan).

We turned to the NMR spectroscopy for structural characterization of the protein. GtgE(17–214) yielded a well-dispersed ^1^H,^15^N-correlation HSQC spectrum characteristic of a well-folded protein (Fig 1A). We also recorded the ^15^N-HSQC spectrum of the previously crystallized GtgE(79–214). Overlay of the HSQC spectra showed deletion of the N-terminal residues led to chemical shift changes for a large number of signals (Fig 1B). The longer fragment displays signals in the spectral regions that commonly contain signals from ß-strands and turns. These features indicate that the N-terminal residues 17–78 are structured and that the absence of this region causes some conformational changes in the remainder of the protein. Attempts to express the N-terminal GtgE(14–79) fragment were not successful suggesting that this region does not fold on its own.

We determined the sequence-specific assignments of the NMR signals using three-dimensional heteronuclear experiments on a ^13^C,^15^N-labeled sample of GtgE(17–214). Due to heterogeneity of the signals and overlap in the middle part of the spectra, we were able to assign backbone signals for only 50% of the residues. Additionally, GtgE showed concentration-dependent tendency to aggregate limiting concentration of NMR samples. These shortcomings precluded us from determination of the NMR solution structure of the protein. Nevertheless, comparison of the NMR assignments with the random coil chemical shifts pointed to a presence of two mobile regions in the N-terminal part of GtgE (S1 Fig). Based on this information we have prepared two deletion mutants GtgE(17–214, Δ33–40, C45S) and GtgE(17–214, Δ59–68, C45S). Expression and purification of these mutants resulted in high yields of both constructs, suggesting that the mutations did not disrupt the structure. Both mutants were screened for crystallization, and the GtgE(17–214, Δ33–40, C45S) mutant produced large crystals diffracting to high resolution.

### Structure of GtgE

**Fig 2:**
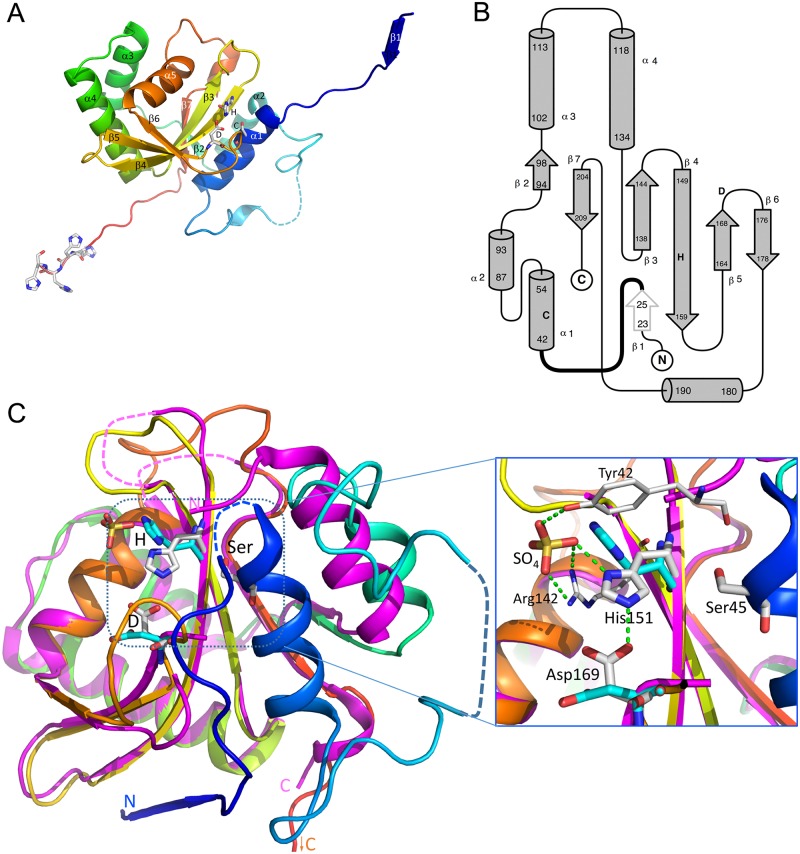
Structure of GtgE(17–214, Δ33–40, C45S). (A) Cartoon representation with the active site residues shown in a stick representation and marked with one-letter code. Position of Ser45 is marked with letter C to indicate that this residue is a cysteine in the wild type GtgE. The molecule is colored in rainbow, from blue at the N-terminus to red at the C-terminus. The disordered segment is shown by a dashed line. The extended termini contact symmetry-related molecules in the crystal. The strand ß1 extends ß-sheet of the neighboring molecule. The catalytic residues and the four C-terminal histidines are shown in a stick mode. Secondary structure elements are labeled. This and subsequent figures were prepared with PyMol (www.pymol.org). (B) The topology diagram of GtgE. The location of catalytic residues is marked with letters C, H and D. (C) The superposition of GtgE (colored rainbow, this work) and the GtgE(79–214) fragment (magenta, PDB code 4MI7, [22]). The ~20 swapped N-terminal residues of the symmetry related molecule are depicted here to show the structure of an intact GtgE (see Fig 3C). The N- and C-termini are marked, the active site residues, Ser45 (Cys45 in wild type GtgE), His151 and Asp169 are shown in stick mode and colored white for GtgE and cyan for GtgE fragment. The inserts on the right shows the expanded view of the active site as observed in our structure. A sulfate molecule is bound near the catalytic histidine and forms hydrogen bonds with its ND1 atom as well as with Tyr42 and Arg142 sidechains. In the presence of a substrate the His151 sidechain would flip by 180° to form hydrogen bond between its ND1 nitrogen and SG of Cys45. Green dashed line indicates the hydrogen bond between His151 and Asp169 observed in the GtgE but not in the GtgE(79–214) fragment.

Our structure of GtgE shows that the enzyme has a papain-like fold, composed of a slightly bent seven-stranded ß-sheet with two ¶-helices on the convex side and three on the concave side (Fig 2A). The helices on the convex side are placed similarly to other papain-like cysteine proteases, while only one of the concave side helices aligns well with other papain-like structures. The other two are either shifted or are not present in other structures. The active site is located on the convex side of the ß-sheet, near the N-terminal end of strand ß4, which carries His151 (Fig 2B and S2 Fig). Asp169 is located at the end of strand ß5 and Ser45 (cysteine in the wild type enzyme) is near the N-terminus of helix α1. The substrate-binding site extends along the convex surface of the ß-sheet passing between helix α1 and tips of strands ß3 and ß4 (Fig 2A and 2B). GtgE has a size similar to papain and lacks the extensions observed in larger papain-like cysteine proteases, e.g. cathepsins. Comparison with the previously determined GtgE(79–214) fragment is shown in Fig 2C. In addition to more disordered loops in the latter, the largest difference is in helix α2 (magenta in Fig 2C), which is shifted away to make space for helix α1 (absent in GtgE(79–214)) and has a kink near its C-terminus.

**Fig 3:**
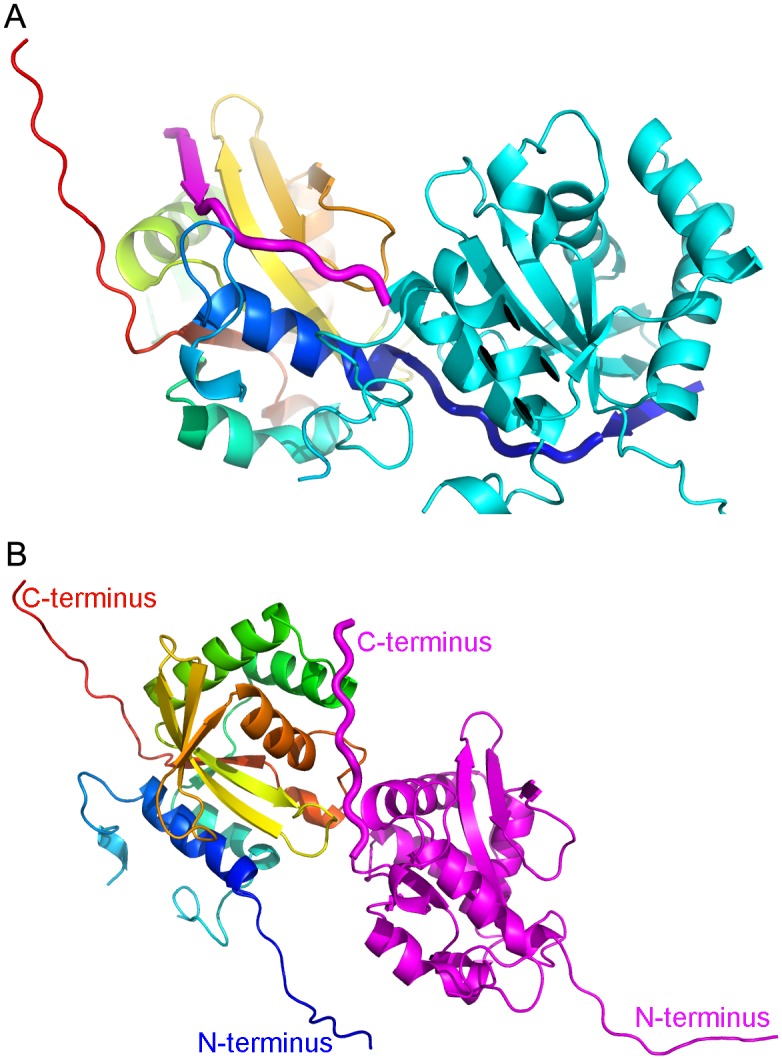
Interactions of the N- and C-terminal segments with neighboring molecules. (A) Segments 17–32 are swapped between two molecules related by 2-fold symmetry and are shown as thick ribbon with residues 23–26 in ß-strand conformation. One molecule is painted rainbow, the other is cyan with the N-terminus colored magenta. (B) The extended C-terminus containing His-tag of magenta molecule contacts a symmetry-related molecule (rainbow colored, N-terminus in blue to C-terminus in red). The extended C-terminus is shown as thick ribbon. These interactions connect molecules into a long chain. (C) A model of GtgE reconstructed by re-swapping the N-terminal segment. The residues 40–214 of molecule A were connected to the swapped segment 17–32 of molecule B through a short linker.

In our GtgE crystal structure, the first five residues of the construct, which starts at Thr17, are disordered. Both the N- and C-terminal segments are extending away from the core of molecule and form intimate contacts with neighboring molecules (Fig 3A and 3B). The ~20 N-terminal residues follow a groove within the second molecule, which is located on the convex side of the ß -sheet, and the extended chain makes contacts with strands ß3 and ß4 as well as helices α1 and α4. At the same time, the N-terminal segment of the second molecule follows the equivalent groove in the first molecule. This groove is the usual location of the N-terminal polypeptide within the papain fold. Thus the N-terminal ~20 residues complete the papain fold of the neighboring molecule, which in turn donates this segment back to the first molecule. Through this swap of their N-termini the two molecules form a tight dimer in the crystal. Since the molecular weight determined by the SEC-MALS of 25.5 kDa indicated that the protein is predominantly a monomer in solution (S3 Fig), this swap is likely the crystallization artefact. Nevertheless, we can reconstruct the structure of the GtgE monomer by swapping back the N-termini and reconnecting residue Tyr42 to Ser32 following the conformation of this loop in papain (Fig 3C). The second segment identified from the HSQC spectrum as mobile, residues 59–68, is indeed partially disordered in the crystal and residues 66–71 were not modeled due to lack of convincing electron density.

**Fig 4:**
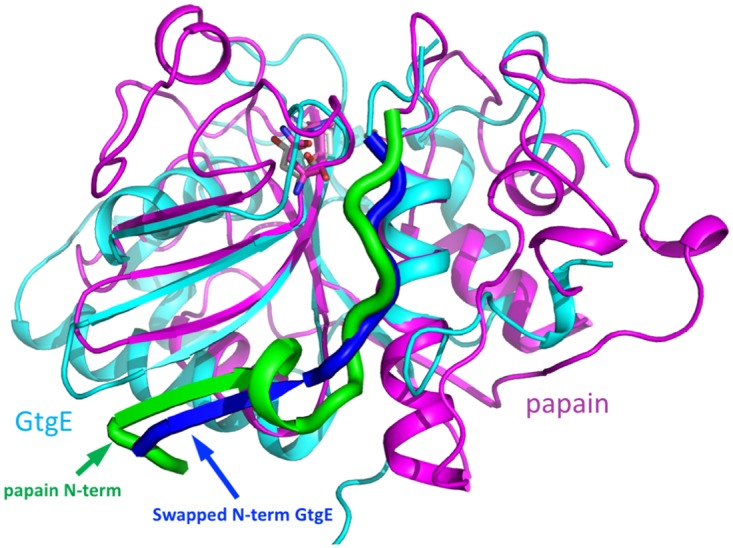
Superposition of GtgE (cyan) and papain (magenta). Papain structure used for the comparison has PDB code 3CVZ [33]. (A) The N-terminus of papain is shown as a thick green ribbon. The swapped N-terminus of GtgE from a neighboring molecule is shown as a thick blue ribbon. GtgE and papain superimpose well within the core of the fold, enclosed within the blue circle. (B) Close-up of the catalytic triads Cys-His-Asp/Asn in this superposition. The Ser45 of GtgE was replaced here by a cysteine and the sidechain of His151 was rotated by 180°. The catalytic residues are shown in stick representation, magenta carbons in papain, white carbons in GtgE.

The core of GtgE, composed of the ß-sheet and surrounding α-helices, superimposes with several other papain-like proteases with a root-mean-squares deviation ranging between 1.5–1.8 Å for ~65 Cα atoms. The superposition with papain is shown in Fig 4A. The active site of GtgE, consisting of Cys45-His151-Asp169, is in a very similar arrangement to that found in papain (PDB code 3CVZ, [33]) (Fig 4B) and other papain-like enzymes. However, the conformation of the triad side chains would have to adjust slightly to form the hydrogen bond pattern expected in the active state of the enzyme (Fig 4B). That would likely involve a small shift of the loop between strands ß3 and ß4 preceding His151.

Comparison with papain and other papain-like enzymes suggests that helix α1 in GtgE is longer by one turn on the N-terminal end and starts four residues earlier. This additional turn of the helix enters the area corresponding to the classic substrate-binding site of papain-like enzymes and partially blocks it. This occlusion of the substrate binding site might have contributed to our lack of success in capturing the GtgE(17–214, Δ33–40, C45S)-peptide complex either by soaking or by co-crystallization. The Δ33–40 deletion is unlikely to be the cause of the lack of peptide binding since the GtgE(17–214) construct showed at most very weak binding of the peptide as measured by NMR. Moreover, GtgE(2–228), GtgE(14–224), GtgE(17–224) and GtgE(17–214, Δ33–40) display similar, albeit low catalytic activity.

Interestingly, the C-terminus also extends away from the GtgE core (Fig 3B). Of the 6xHis tag, four histidines are well defined in the electron density. This region contributes significantly to crystal contacts helping the formation of an extended row of molecules (Fig 3B).

### Protease activity of GtgE constructs

**Fig 5:**
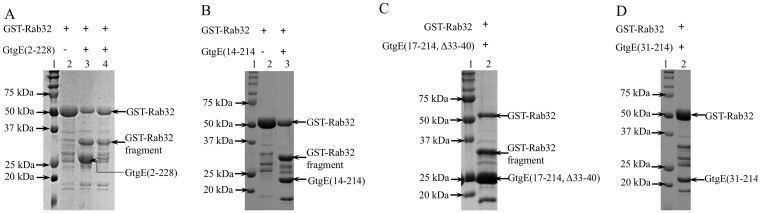
Superposition of GtgE (cyan) and papain (magenta). Papain structure used for the comparison has PDB code 3CVZ. **(A**) Cleavage of GST-Rab32 by GtgE(2–228). Lane 1: protein markers, lane 2: GST-Rab32 fraction. The protein was only ~80% pure, other unidentified lower molecular weight bands were present in small amounts, lane 3: Cleavage of GST-Rab32 by GtgE(2–228) mixed in 2:1 (substrate:enzyme) molar ratio, lane 4: As lane 2 but 20:1 ratio of Rab32:GtgE(2–228). (B) Cleavage of GST-Rab32 by GtgE(14–214), lane 1: protein markers, lane 2: partially purified GST-Rab32 fraction, lane 3: Cleavage of GST-Rab32 by GtgE(14–228) in 2:1 molar ratio. (C) Cleavage of GST-Rab32 by GtgE(17–214, Δ33–40). Lane 1: protein marker,s lane 2: Cleavage of GST-Rab32 by GtgE(17–214, Δ33–40) in 2:1 molar ratio; **D)** GST-Rab32 in the presence fo GtgE(31–214). Lane 1: protein marker, lane 2: GST-Rab32 and GtgE(31–214) in 2:1 molar ratio. The unmarked bands were present in partially purified GST-Rab32 (see panel B lane 2), no band corresponding to the GST-Rab32 degradation product was observed.

To determine if the recombinant proteins retained enzymatic activity, we monitored cleavage of GST-Rab32, which was previously shown to be a GtgE substrate [19]. GtgE cleaves Rab32 after Gly59 resulting in a ~35 kDa GST-Rab32(1–59) fragment [30]. The appearance of this fragment was followed by SDS-PAGE. Addition of His-GtgE(2–228) leads to cleavage of GST-Rab32 and appearance of the 35 kDa band (Fig 5). The activity was relatively low as evidenced by the detection of some intact substrate 2 h after the onset of the reaction. As expected, the GtgE(2–228, H151S) mutant had no detectable activity.

The activity of the GtgE(14–214) construct was comparable to His-GtgE(2–228); however, the shorter GtgE(31–214) showed significantly lower activity. Therefore, we conclude that residues 14–30 are important for the full protease activity of GtgE.

The structure of GtgE allows us to explain the catalytic properties of the N-terminal deletion mutants. Removal of the first ~20 residues does not disturb the overall structure of GtgE as they are not essential for proper folding. We can speculate about the reason for inactivity of the GtgE(31–214) construct. Residues 22–31 are forming intimate contacts with the bulk of GtgE, in particular adding a ß-strand to the C-terminal part of strand ß4 and interacting with the sidechain from helix α4, loop following it and helix α1. The loop following residue 31 and showing hallmarks of disordered segment is likely contributing to the substrate binding and recognition site. The removal of the 1–31 segment would lead to permanent disorder of the loop 32–42 and change the substrate-binding site. It could also affect the positioning of the catalytic Cys45 located just downstream from the mobile loop.

### NMR titrations of GtgE with substrates

In order to test the binding of GtgE substrates, we titrated the ^15^N-labeled GtgE(17–214) with a peptide, RATIGVDFALK, that incorporates the cleavage site of Rab32. The titration was monitored by acquiring ^1^H,^15^N-correlation NMR spectra upon every addition of the peptide. In order to prevent cleavage, the catalytically inactive C45S GtgE mutant was used. No significant spectral changes were observed even at 5-fold molar excess of the peptide, suggesting very weak or no binding (S4 Fig). We hypothesized that the Rab substrates have a GtgE-binding site outside of the cleavage site. We titrated the ^15^N-labeled GtgE(17–214) with the MBP-fused Rab29. Binding of the large protein would normally result in broadening and disappearance of many signals of the labeled protein due to lower tumbling rates of the resulting complex. No appreciable signal broadening was observed at 2:1 molar ratio of GtgE to MBP-Rab29.

The low activity of all the constructs observed in the *in vitro* assays (with the exception of GtgE(31–224), which is inactive) and partial occlusion of the putative substrate binding site by the extension of helix α suggest a participation of an additional factor or factors within the host cell that are necessary to activate GtgE for efficient recruitment and cleaveage of Rab substrates near the SCV membrane. Interaction of GtgE with such activator would cause some conformational rearrangement around the putative substrate binding site, possibly unwinding of the first turn of helix α1 and small rearrangement of the catalytic residues to form a competent active site. Similar activation was observed, for example, for the effector kinase OspG from *Shigella flexneri* upon binding of ubiquitin, and this effect was even stronger upon binding of ubiquitin ligase UbcH5 or UbcH7 conjugated with ubiquitin [34, 35].

## Conclusions

We have used NMR spectroscopy to identify internal flexible regions of GtgE and use this information to design mutants in which these regions were deleted individually. One of these deletion mutants, Δ33–40, led to well diffracting crystals and the protein retained activity comparable with the wild type enzyme. The protein displays papain-like fold and contains Cys-His-Asp catalytic triad but the triad is slightly misaligned. Using a physiological substrate, Rab32, we observed low activity *in vitro* and very weak binding to a peptide encompassing the cleavage site of its physiological substrate. We conclude that the function of GtgE *in vivo* as a proteolytic enzyme is likely dependent on other factor(s), such as a protein partner or interactions with the SCV membrane, which cause a realignment of the catalytic triad to its active conformation stimulating GtgE activity *in vivo*.

## Supporting Information

S1 Fig Design of GtgE deletion mutants. **A)** Secondary structure prediction using Jpred suggests two regions in the N-terminal part of GtgE with no predicted secondary structure. The areas targeted by mutagenesis are highlighted with boxes; **B)** Plot of Ca chemical shift differences from random coil values shows an absence of defined secondary structure in 36–40 and 63–79 regions. Secondary structure as seen in the crystal structure is shown above the plot. The residues without bars were not assigned in the NMR spectra due to overlaps or missing signals.
(TIF)

S2 Fig Omit electron density map of GtgE catalytic residues. Cys45 was mutated to serine in the crystallized construct. Electron density is contoured at 1 σ from the 2F_O_-DF_C_ omit map.
(TIF)

S3 Fig Molecular Mass determined by Multi-Angle Light Scattering and Refractive Index measurement. The protein was loaded on high resolution size exclusion column and eluted in the same buffer system the protein was prepared for crystallization.
(TIF)

S4 Fig Overlay of HSQC spectra of 0.2 mM ^15^N-labeled GtgE(14–214) in 20 mM HEPES pH 7.5, 100 mM NaCl in the absence (red) and presence (blue) of Rab-derived peptide at 1:5 molar ratio of protein to peptide. (TIF)
