# Supplementary figures and images for: Crystal Structure of the Salmonella Typhimurium Effector GtgE
Source: PLoS One. 2016 Dec 6;11(12):e0166643. doi: 10.1371/journal.pone.0166643 (PMC5140068; doi:10.1371/journal.pone.0166643)

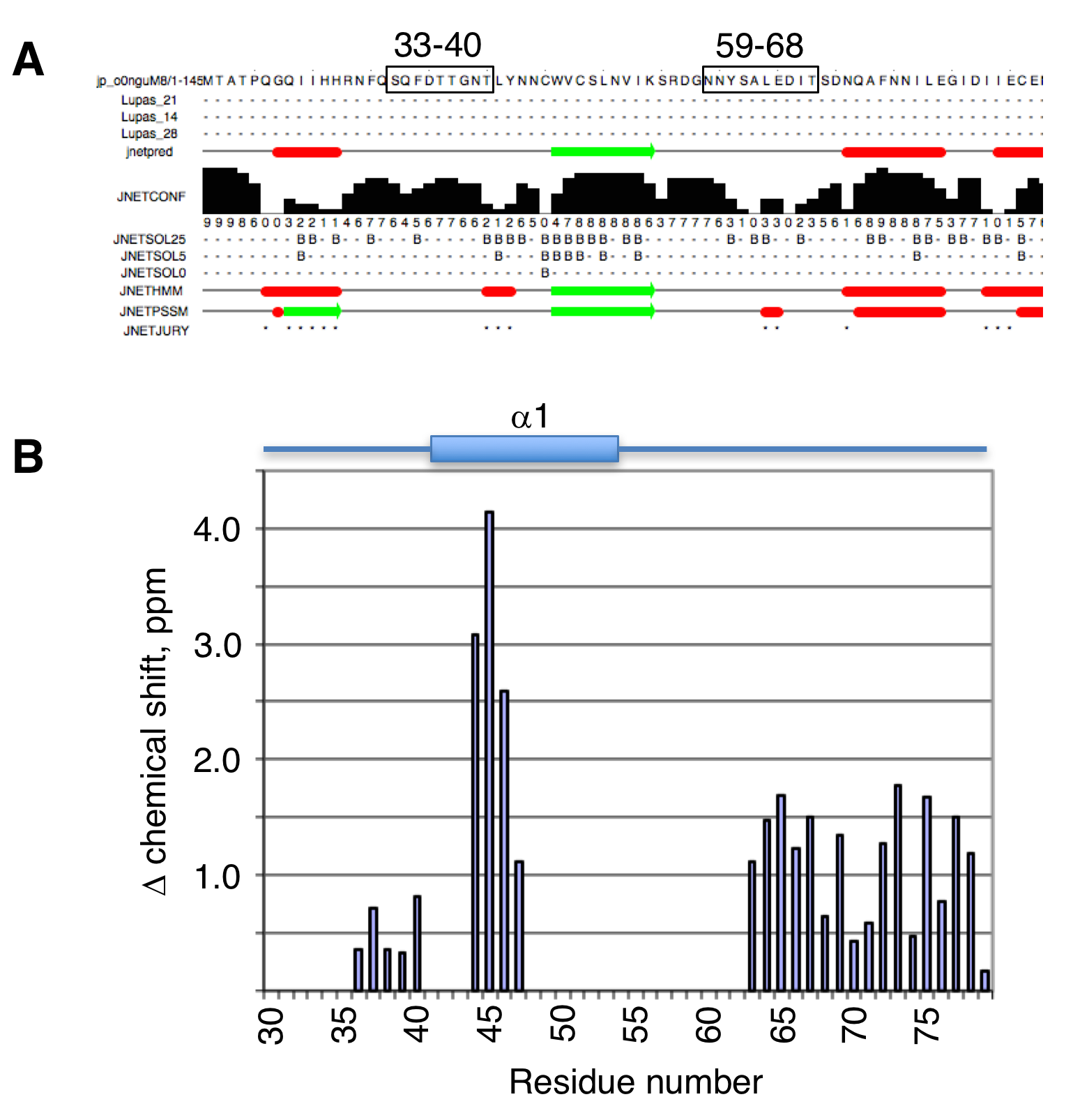

Supplement: S1 Fig — A) Secondary structure prediction using Jpred suggests two regions in the N-terminal part of GtgE with no predicted secondary structure. The areas targeted by mutagenesis are highlighted with boxes; B) Plot of Ca chemical shift differences from random coil values shows an absence of defined secondary structure in 36–40 and 63–79 regions. Secondary structure as seen in the crystal structure is shown above the plot. The residues without bars were not assigned in the NMR spectra due to overlaps or missing signals. (TIF) [file pone.0166643.s001.tif]

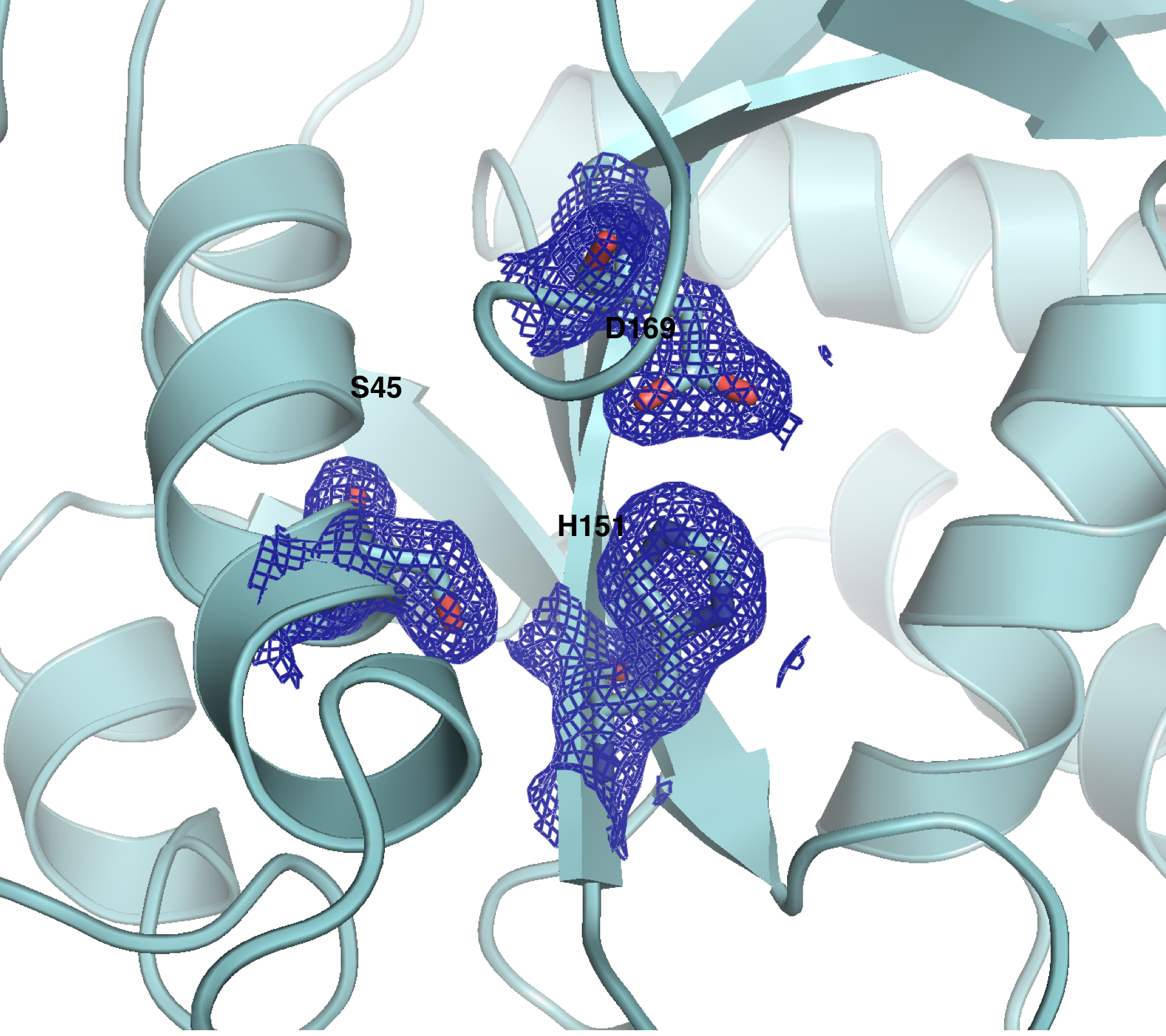

Supplement: S2 Fig — Cys45 was mutated to serine in the crystallized construct. Electron density is contoured at 1 σ from the 2FO-DFC omit map. (TIF) [file pone.0166643.s002.tif]

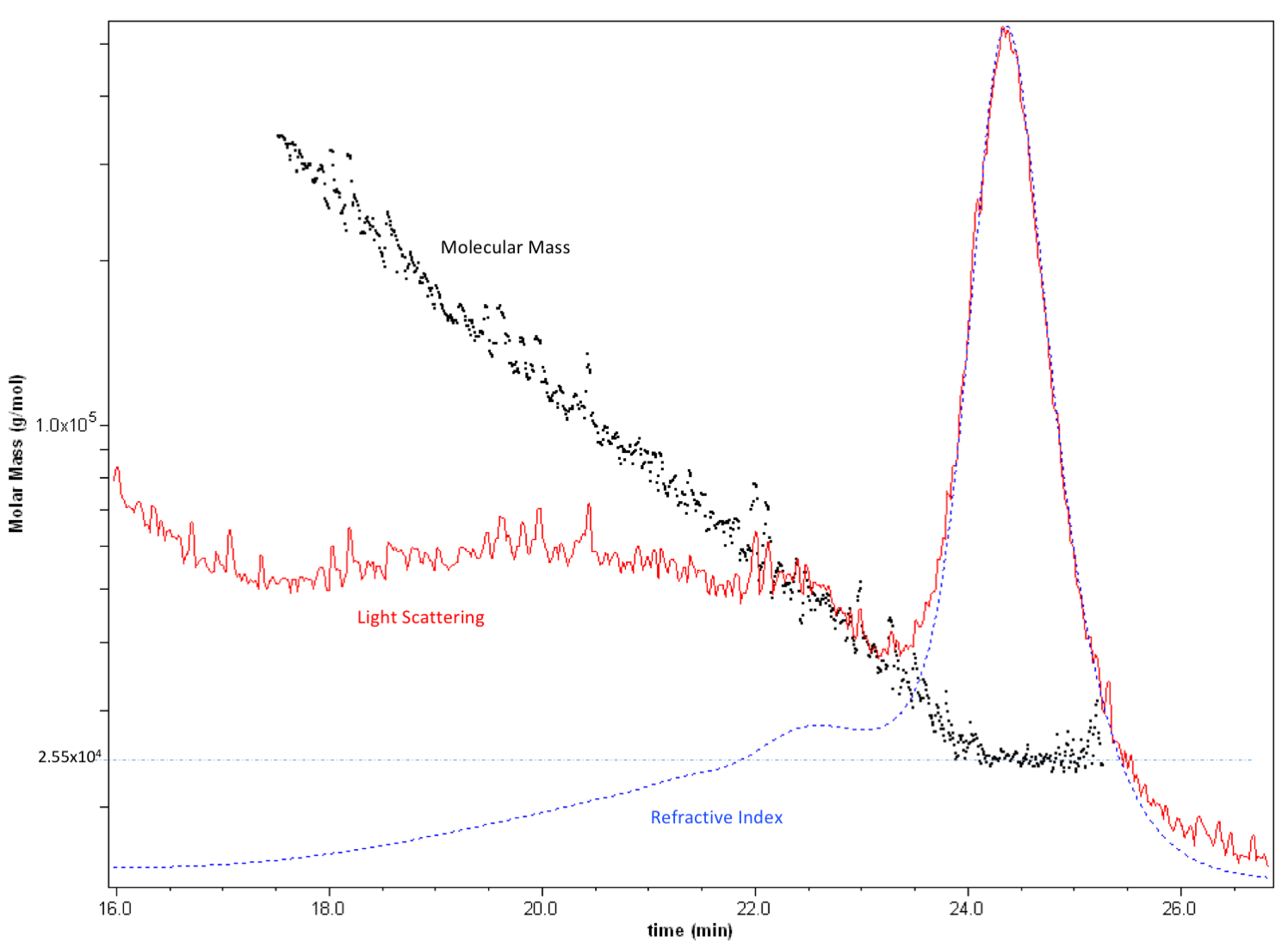

Supplement: S3 Fig — The protein was loaded on high resolution size exclusion column and eluted in the same buffer system the protein was prepared for crystallization. (TIF) [file pone.0166643.s003.tif]

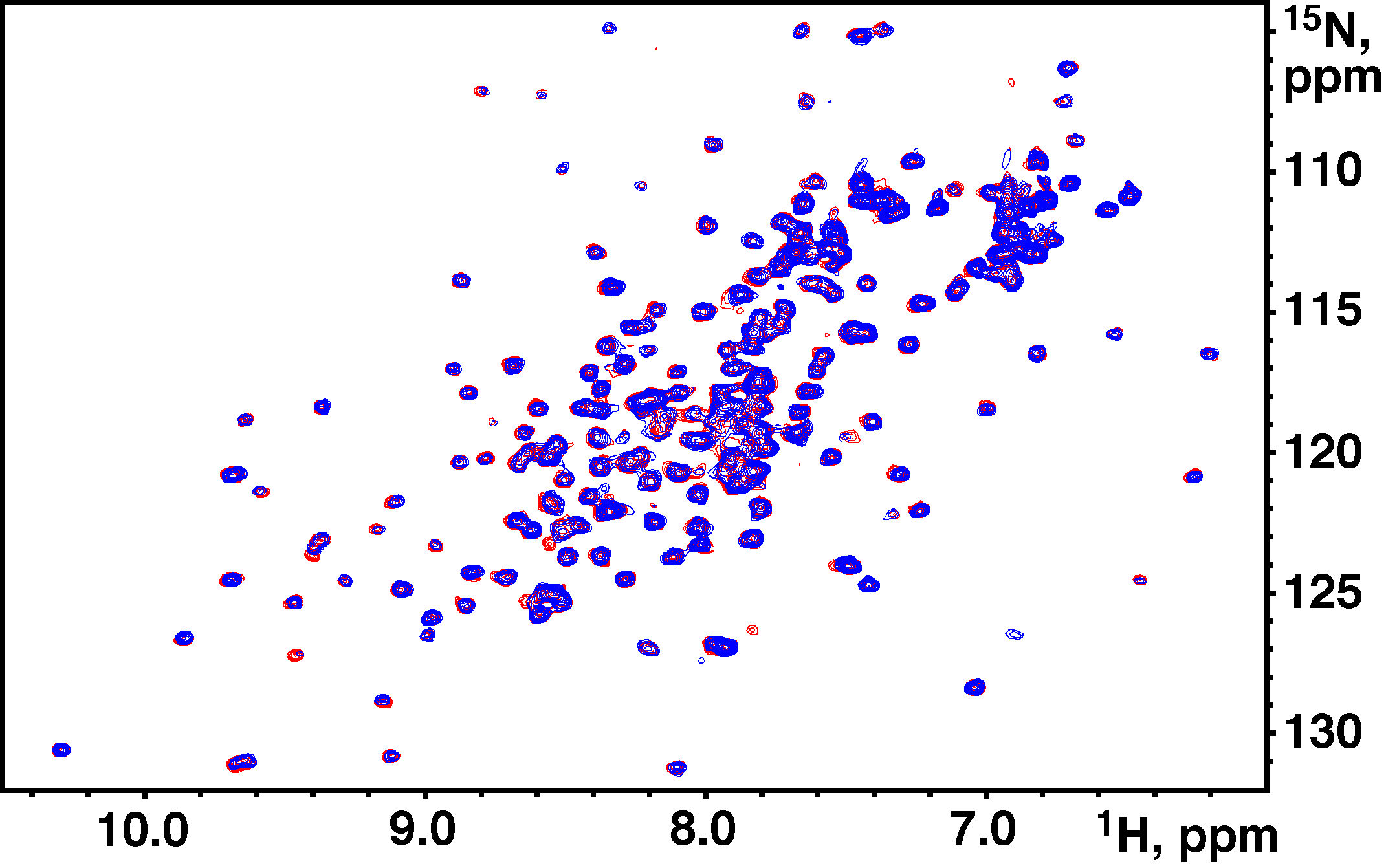

Supplement: S4 Fig — (TIF) [file pone.0166643.s004.tif]
